# Hydrogel Walkers with Electro-Driven Motility for Cargo Transport

**DOI:** 10.1038/srep13622

**Published:** 2015-08-28

**Authors:** Chao Yang, Wei Wang, Chen Yao, Rui Xie, Xiao-Jie Ju, Zhuang Liu, Liang-Yin Chu

**Affiliations:** 1 School of Chemical Engineering, Sichuan University, Chengdu, Sichuan 610065, China; 2 State Key Laboratory of Polymer Materials Engineering, Sichuan University, Chengdu, Sichuan 610065, China

## Abstract

In this study, soft hydrogel walkers with electro-driven motility for cargo transport have been developed *via* a facile mould-assisted strategy. The hydrogel walkers consisting of polyanionic poly(2-acrylamido-2-methylpropanesulfonic acid-*co*-acrylamide) exhibit an arc looper-like shape with two “legs” for walking. The hydrogel walkers can reversibly bend and stretch via repeated “on/off” electro-triggers in electrolyte solution. Based on such bending/stretching behaviors, the hydrogel walkers can move their two “legs” to achieve one-directional walking motion on a rough surface via repeated “on/off” electro-triggering cycles. Moreover, the hydrogel walkers loaded with very heavy cargo also exhibit excellent walking motion for cargo transport. Such hydrogel systems create new opportunities for developing electro-controlled soft systems with simple design/fabrication strategies in the soft robotic field for remote manipulation and transportation.

Stimuli-responsive hydrogels with controllable volume/shape changes in response to external stimuli such as electric field^1^,^2^,^3^, temperature^4^,^5^,^6^, pH^7^,^8^, humidity^9^,^10^,^11^, and solvent composition^12^,^13^, show remarkable potential for developing biomimetic soft robotics for controllable triggered manipulation such as actuation, locomotion, and cargo transportation^14^,^15^,^16^,^17^,^18^,^19^,^20^,^21^,^22^,^23^. Among these hydrogels, electro-responsive hydrogels are of particular interests, because the electric stimulus can be simply and remotely controlled with controllable strength and direction, and rapid and precise "on/off" triggering switch, as compared with other stimuli. These hydrogels allow electric-field-induced swelling, shrinking, or bending, which creates opportunities for transforming electric energy into mechanical motion via elaborately designing the hydrogel structures^24^,^25^,^26^,^27^,^28^. Therefore, fabrication of soft robotics from electro-responsive hydrogel materials are of significant importance for developing intelligent systems with simple control strategy for remote manipulation such as self-locomotion and cargo transportation.

Typically, electro-responsive hydrogels are made of polyelectrolyte networks that contain ionic groups. Under electric fields, the polyelectrolyte networks can achieve asymmetric swelling/shrinking volume changes as well as bending behaviors, due to the osmotic pressure difference induced by electric-field-driven ion motion. Based on the electro-induced volume/shape changes, electro-responsive hydrogels are widely used for engineering soft chemo-mechanical systems, such as artificial crawler^2^, artificial muscle^29^,^30^,^31^, mechanical hand^25^, and artificial fish^32^. Looper-like hydrogel crawlers with hooks hanging on a ratchet track, are developed from poly(2-acrylamido-2-methylpropanesulfonic acid) (PAMPS) hydrogel^13^. Under electric field, one side of the PAMPS hydrogel that faces the anode can form complex with the positively-charged surfactant n-dodecylpyridinium chloride from external solution, leading to asymmetric volume contraction and bending of the hydrogel towards the anode. Thus, with oscillating electric field, the hydrogel crawlers can achieve looper-like walking under water by repeatedly stretching and bending. However, their movement requires existence of surfactants in external aqueous media, and demands hooks to hang on the ratchet tracks, thus limiting their broad application. Alternatively, hydrogel actuators that can walk on flat elastomer substrates are developed by electrically and chemically combining one cationic gel leg with another anionic gel leg^33^. The two legs can deform in opposite directions under electric field, and thus enable the hydrogel actuators to achieve unidirectional motion. However, the dual-leg hydrogel actuator requires troublesome processes for combining the two legs. Moreover, although these electro-responsive hydrogels can exhibit electro-induced motion, further study on the performances of electro-responsive hydrogels for loading cargo are still needed for utilizing such systems as soft robotics for remote-controllable cargo transport. Therefore, a simple approach for fabricating soft hydrogel systems with electro-driven motility for cargo transport is highly still desired.

In this study, we report on a facile strategy for fabricating hydrogel walkers with electro-driven motility for cargo transport. The hydrogel walkers consist of crosslinked polyanionic poly(2-acrylamido-2-methylpropanesulfonic acid-*co*-acrylamide) (poly(AMPS-*co*-AAm)) networks with an arc looper-like shape. The hydrogel walkers can reversibly bend and stretch via repeated “on/off’ electrical fields in electrolyte solution to achieve one-directional walking motion on a rough surface. Furthermore, the hydrogel walkers can be used to load very heavy cargo for efficient one-directional transportation. Such hydrogel systems are promising as soft robotics for cargo transport, and also show great potential for developing remotely-controllable soft systems such as artificial muscles and actuators.

## Results

### Fabrication of Poly(AMPS-*co*-AAm) Hydrogel Walkers

**Figure 1:**
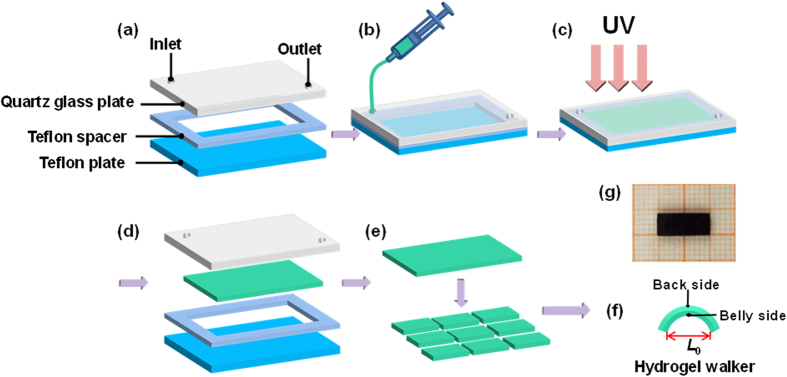
Strategy for fabricating the poly(AMPS-*co*-AAm) hydrogel walkers. (**a–f**) Fabrication process. First, a mould is assembled with a Teflon plate, a Teflon spacer and a quartz glass plate (**a**). Then, monomer solution is injected into the mould (**b**) for UV-initiated polymerization (**c**). Next, the prepared hydrogel was taken out from the disassembled mould (**d**), and then cut into strips (**e**) as hydrogel walkers (**f**). (**g**) Optical photo that showing a hydrogel walker at stretch state. All of the drawing components in the figure are created by Chao Yang.

The fabrication procedure of poly(AMPS-*co*-AAm) hydrogel walkers is shown in Fig. 1. For fabricating hydrogel walkers with looper-like shape, a mould is assembled from a quartz glass plate, a Teflon plate and a Teflon spacer for hydrogel synthesis (Fig. 1a). Next, monomer solution containing 2-acrylamido-2-methylpropanesulfonic acid (AMPS), acrylamide (AAm), *N*,*N*’-methylenebisacrylamide (MBAA), and 2-oxoglutaricacid (KGA) is injected into the mould (Fig. 1b), and then polymerized under UV-initiated irradiation to convert the monomer solution into crosslinked hydrogel (Fig. 1c). After that, the synthesized hydrogel is taken out of the dissembled mould (Fig. 1d), and washed with water. At last, the hydrogel is cut into uniform strips (Fig. 1e) as the hydrogel walkers (Fig. 1f), and dyed with methylene blue for further investigations (Fig. 1g).

**Figure 2:**
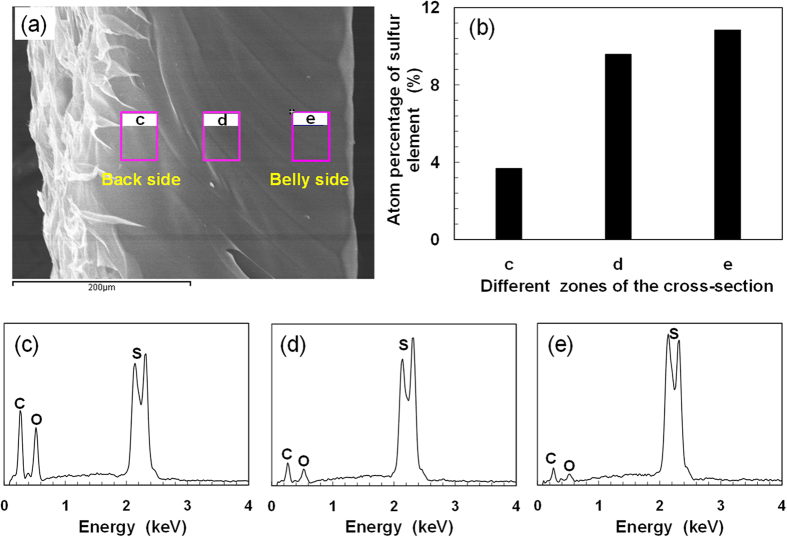
Chemical composition analysis of the cross-section of poly(AMPS-*co*-AAm) hydrogel walker. (**a**) SEM image showing the cross-section of poly(AMPS-*co*-AAm) hydrogel walker. (**b**) Distribution of sulfur element at different zones of the cross-section. (**c–e**) EDS spectra of the hydrogel at zone (**c**), zone (**d**) and zone (**e**).

For the poly(AMPS-*co*-AAm) hydrogel walkers, the AMPS acts as the electro-responsive moiety due to its strongly ionizable sulfonate group; while the AAm serves as the copolymer to improve the mechanical strength of the fragile PAMPS hydrogel. The effects of the monomer ratio on the electro-responsive property and the mechanical strength of the hydrogels are shown in Figs S1 and S2, with the total molarity of AMPS and AAm fixed at 1 M. Under voltage of 25 V, the hydrogel walkers with 0.8 M and 0.7 M AMPS show a dramatic and relatively uniform bending behavior with a very short time (<~6 s) to reach 100% of the degrees of bending deformation (*ε*), as compared to those with 0.6 M and 0.5 M AMPS (Fig. S1). However, the hydrogel walker with 0.8 M AMPS shows weaker mechanical strength than the one with 0.7 M AMPS (Fig. S2). Thus, AMPS content of 0.7 M is chosen for fabricating the hydrogel walker with both fast response and good mechanical strength for further study. In the mould, the AMPS and AAm monomers are polymerized under UV irradiation from the quartz glass side. Due to the attenuation of UV light strength along the irradiation distance, there exists a gradient distribution of crosslinkage of hydrogel networks along the spacer height. Near the quartz glass surface side where the UV light strength is higher, more densely crosslinked hydrogel networks can be formed during UV-polymerization; while near the Teflon surface side where the UV light strength is lower, a more loosely crosslinked networks are formed. The anisotropic networks of the hydrogel can lead to asymmetric swelling/deswelling volume transition behaviors. As a result, when immersed in water, the hydrogel walker can slightly bend to the more densely crosslinked side, exhibiting an initial looper-like shape (Fig. 1f). Here we define the convex of the arc-shaped hydrogel walker as the “back side” (light green color) and the concave as the “belly side” (dark green color), just as those of crawlers in nature. The chemical composition of the hydrogel walker is characterized by FT-IR (Fig. S3), which confirms the successful copolymerization of the AMPS and AAm for fabrication of poly(AMPS-*co*-AAm) hydrogel. To investigate the crosslinkage of hydrogel walker, the distribution of sulfur element at the cross-section of hydrogel walker, which is only contained in the AMPS monomer, is studied by SEM that coupled with Energy Dispersive Spectrometer (EDS) (Fig. 2). Three different zones at the cross-section of the hydrogel walker (Fig. 2a) are selected for detection of sulfur element. The increase in the atomic percentage of sulfur element (Fig. 2b) from the "back side" to the "belly side" (Fig. 2c–e) indicates the gradient distribution of crosslinkage along the cross-section of the hydrogel walker. Such results confirm that the "belly side" possesses more densely crosslinked networks than the "back side".

### Electro-Induced Bending Behaviors of Hydrogel Walkers

**Figure 3:**
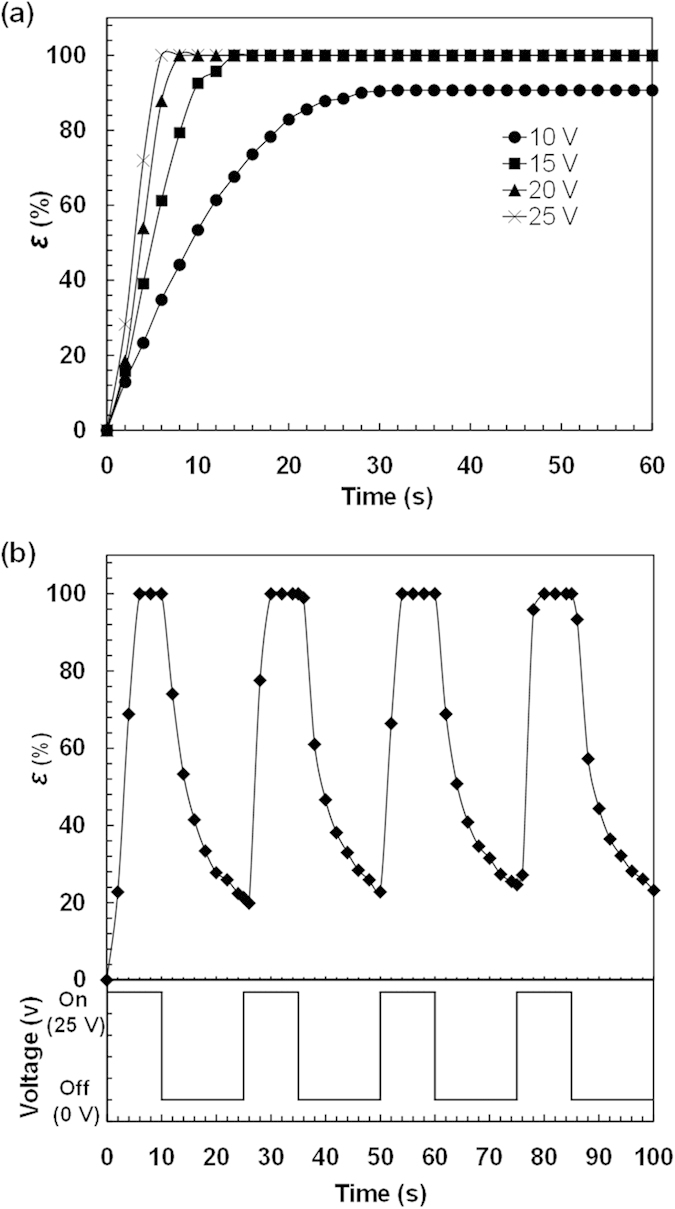
Electro-induced bending behavior of the hydrogel walker. (**a**) Effect of applied voltage on the degree of bending deformation (*ε*) of the hydrogel walker. (**b**) Reversible bending/stretching behavior of the hydrogel walker under repeated "on/off" electric fields.

The poly(AMPS-*co*-AAm) hydrogel is a polyanionic hydrogel that can bends toward the cathode in electrolyte solutions quickly and significantly. Thus, the electro-induced bending behaviors of the hydrogel walkers are investigated by immersing them between two horizontally-placed carbon electrodes in NaCl solution, with the belly side and the back side respectively facing the cathode and the anode (Fig. S4). As shown in Fig. 3a, when applied with voltages of 15 V, 20 V and 25 V, the hydrogel walkers can bend toward the belly side as well as the cathode side, with the degrees of bending deformation (*ε*) reaching 100% as compared with the one under voltage of 10 V (*ε* = ~90.69%). Meanwhile, with increasing the voltage from 10 V to 25 V, the time required for the hydrogel walker to reach the equilibrium bending deformation reduces from ~32 s to ~6 s. The results indicate that the poly(AMPS-*co*-AAm) hydrogel walkers can achieve faster and more significant bending behaviors with increasing voltage. Therefore, we can control the bending behaviors of the hydrogel walkers by regulating the applied voltage.

The reversibility and repeatability of the bending behaviors are investigated by applying the poly(AMPS-*co*-AAm) hydrogel walkers under 25 V voltage for fast actuation. As shown in Fig. 3b, when turning on the electric field for 10 s, the hydrogel walker can reach 100% degree of bending deformation; when turning off the electric field for 15 s, the hydrogel can reach ~20% degree of bending deformation at the end of each cycle, showing well-controlled electro-driven stretching. The results demonstrate the excellent reversible and repeatable bending/stretching behaviors of the poly(AMPS-*co*-AAm) hydrogel walkers in response to electric field.

### Electro-Driven Walking Behaviors of Cargo-Loaded Hydrogel Walkers

**Figure 4:**
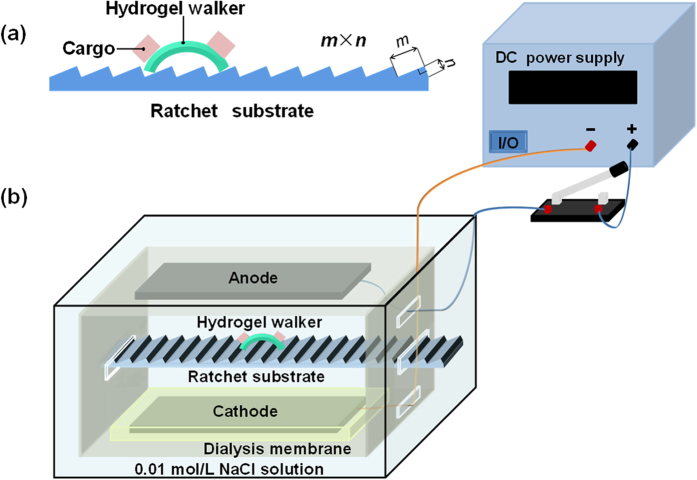
Setup for achieving and observing the electro-driven motility of cargo-loaded hydrogel walker. (**a**) A ratchet substrate used for the walking motion of cargo-loaded hydrogel walker. (**b**) Equipment setup for investigating the electro-driven motility. All of the drawing components in the figure are created by Chao Yang.

The poly(AMPS-*co*-AAm) hydrogel walkers with reversible and repeatable bending/stretching behaviors can achieve one-directional walking motion by applying repeated electrical triggers. To investigate such electro-induced walking behaviors, a resin plate with ratchet surface that fabricated by Stereo Lithography Apparatus is used as the rough substrate for the walking (Fig. 4a). The ratchet plate is immersed in 0.01 mol/L NaCl solution and horizontally placed between two carbon electrodes for the walking of hydrogel walker (Fig. 4b). An electric voltage of 25 V is repeatedly applied to the hydrogel walker, with turning on for 10 s and then turning off for 15 s, to induce the reversible and repeatable bending/stretching behaviors for walking. First, ratchet plates with different step sizes (*m* × *n*) are employed to study the effect of ratchet topography on the walking motion of the hydrogel walker. A cargo-loaded hydrogel walker, with cargo weight (*m*_c_) being 25 times that of the walker at dried state (*m*_0_), is placed on four different ratchet plates, with step sizes of 0.4 mm × 0.2 mm, 0.8 mm × 0.2 mm, 0.8 mm × 0.4 mm, and 1.2 mm × 0.2 mm for electro-induced walking (Supplementary Figs S5 and S6). When *m* × *n* = 0.4 mm × 0.2 mm, the distance between adjacent ratchet steps is too small for supporting the legs of hydrogel walker to achieve efficient walking motion (Supplementary Fig. S5a and Movie S1). When increasing *m* from 0.4 mm to 0.8 mm, namely *m* × *n* = 0.8 mm × 0.2 mm, the hydrogel walker can achieve efficient motion on the ratchet plate step by step (Supplementary Fig. S5b and Movie S2). However, when further increase *n* from 0.2 mm to 0.4 mm, namely *m* × *n* = 0.8 mm × 0.4 mm, the ratchet step becomes too high for the foreleg of hydrogel walker to stride over, thus the hydrogel walker gets stuck between adjacent steps (Supplementary Fig. S5c and Movie S3). Alternatively, when *m* × *n* = 1.2 mm × 0.2 mm, there is more space between adjacent steps for the legs of hydrogel walker to stand, thus sliding occurs during the walking, leading to low-efficient motion (Supplementary Fig. S5d and Movie S4). Therefore, due to the most efficient walking motion of the hydrogel walker on ratchet plate with *m* × *n* = 0.8 mm × 0.2 mm (Supplementary Fig. S6), such a ratchet plate is employed for further study of the walking behaviors.

**Figure 5:**
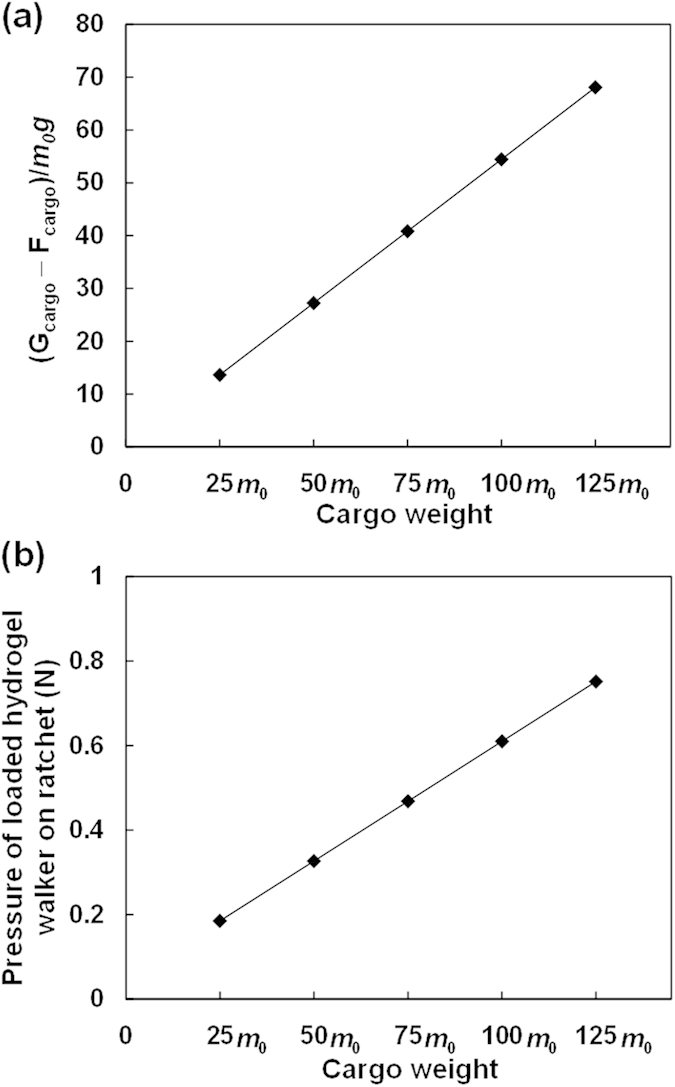
The cargo load of hydrogel walker with different weights of cargo (a), and the pressure on the ratchet (b). (**a**) The ratio of the pressure of cargo (*G*_cargo_ − *F*_cargo_) to the dry mass of hydrogel walker (*m*_0_*g*). (**b**) The pressure of hydrogel walkers loaded with diffierent weights of cargo (*G*_cargo_ + *G*_hydrogel_ − *F*_cargo_ − *F*_hydrogel_) on the ratchet. *G*_cargo_ and *G*_hydrogel_ are the gravity of the cargo and the hydrogel walker respectively, and *F*_cargo_ and *F*_hydrogel_ are the buoyancy of the cargo and the hydrogel walker in water respectively.

**Figure 6:**
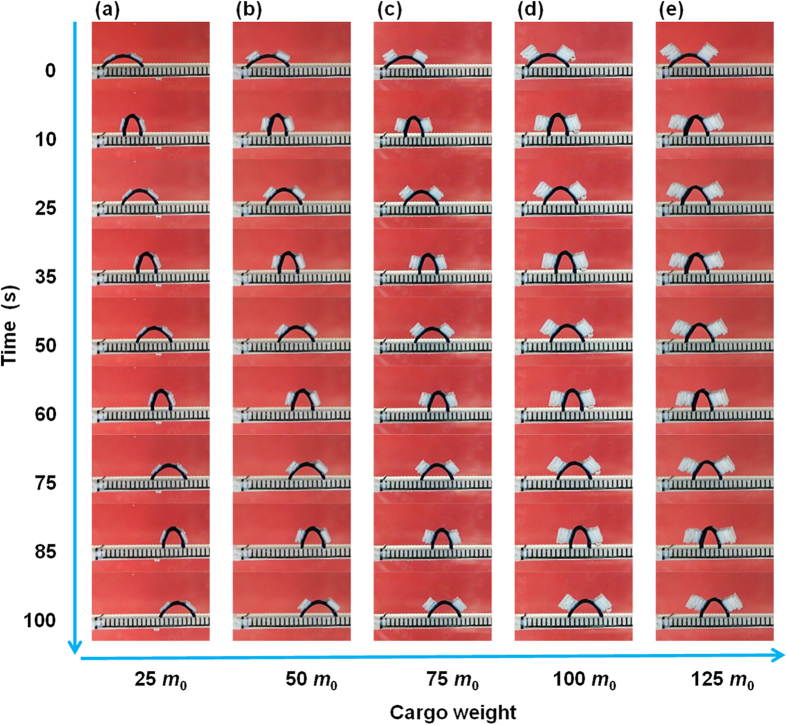
Photographs showing the walking behaviors of hydrogel walkers loaded with different weights of cargo. The cargo weights are: (**a**) 25 *m*_0_, (**b**) 50 *m*_0_, (**c**) 75 *m*_0_, (**d**) 100 *m*_0_, and (**e**) 125 *m*_0_, in which *m*_0_ is the weight of the walker at dried state.

**Figure 7:**
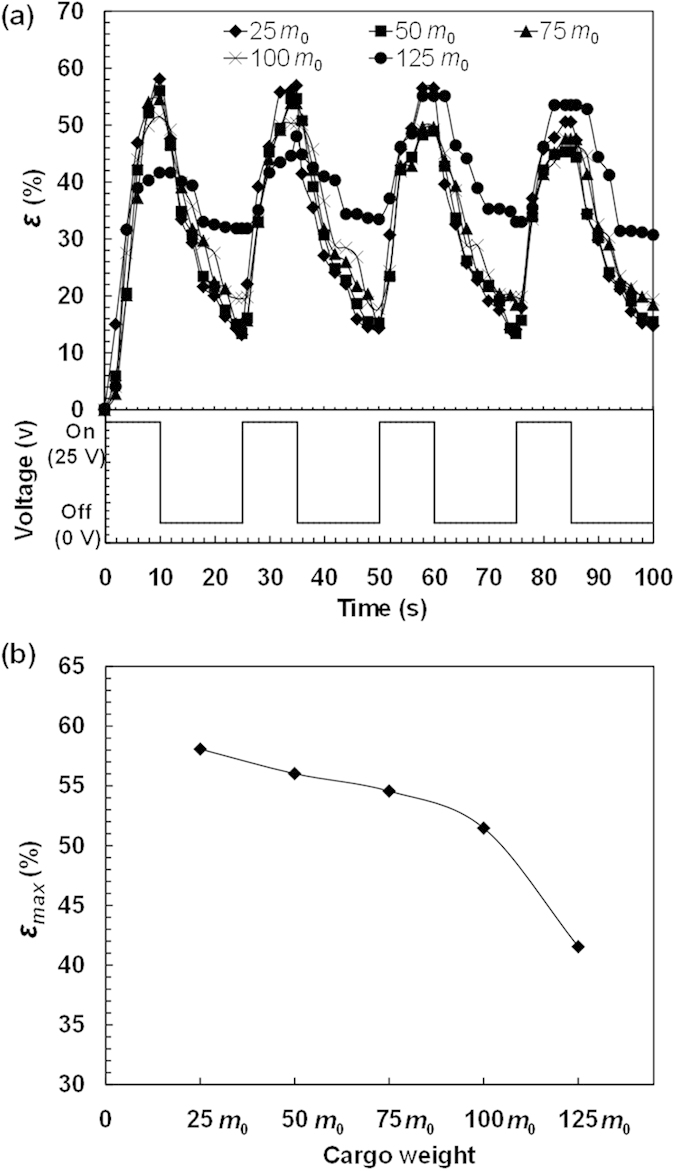
The bending/stretching behaviors of the cargo-loaded hydrogel walkers. (**a**) Bending behaviors of the hydrogel walkers loaded with different weights of cargo during the electro-induced walking. (**b**) Effect of cargo weight on the maximum *ε* value (*ε*_max_) of the hydrogel walkers.

**Figure 8:**
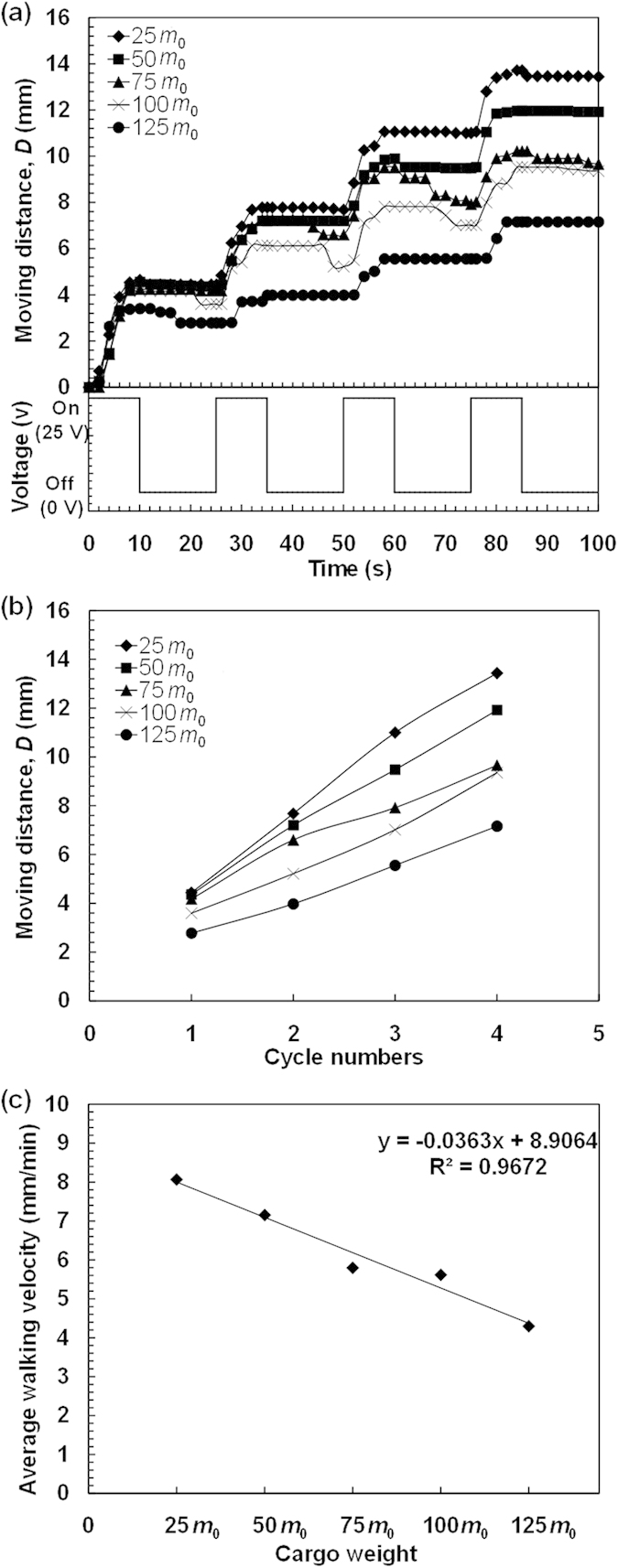
The electro-induced walking behaviors of the cargo-loaded hydrogel walkers. (**a**) Walking behaviors of hydrogel walkers loaded with different weights of cargo. (**b**) Effect of cargo weight on the moving distance for each cycle. (**c**) Effect of cargo weight on the average walking velocity.

To systematically investigate the walking behaviors of the poly(AMPS-*co*-AAm) hydrogel walkers for cargo transport, cargo with different weights of 25 *m*_0_, 50 *m*_0_, 75 *m*_0_, 100 *m*_0_ and 125 *m*_0_, are respectively loaded on the hydrogel walkers for walking (Supplementary Movies S2, S5, S6, S7 and S8). Fig. 5a shows the changes in the ratio of cargo pressure on the hydrogel walker with increasing the cargo weight, which increases from 13.6 to 68.1 with increasing the cargo weight from 25 *m*_0_ to 125 *m*_0_, indicating an increased burden of cargo on the hydrogel walker. Meanwhile, such an increase in the cargo weight also increases the pressure of the loaded hydrogel walker on the ratchet (Fig. 5b), which increases the resistance caused by the surface friction against the movement during the walking. As shown in Figs 6 and 7a, during the walking, all of the hydrogel walkers that loaded with different weights of cargo exhibit excellent reversible and repeatable bending/stretching behaviors upon repeated "on/off" electric trigger. With increasing the cargo weight from 25 *m*_0_ to 125 *m*_0_, the *ε* value of the hydrogel walker decreases from 58.09% to 41.55% (Fig. 7b). The effect of cargo weight on the bending behaviors and resistance can lead to the cargo-loaded hydrogel walkers with different walking behaviors. Fig. 8a shows the time-dependent walking distance of the hydrogel walkers loaded with cargo of different weights upon repeated "on/off" electric triggers. When the cargo weight increases from 25 *m*_0_ to 125 *m*_0_, the walking distance of the hydrogel walker decreases from 13.44 mm to 7.16 mm after fixed numbers of cycles (Fig. 8a). The increased cargo weight restricts the bending behaviors of the hydrogel and increases the resistance, thus leading to decreased walking distance per cycle (Fig. 8b) as well as decreased walking velocity (Fig. 8c). The results show the power of the hydrogel walkers for remotely-controlled transportation of very heavy cargo.

## Discussion

To create an arched hydrogel walker with two legs for walking motion, poly(AMPS-*co*-AAm) hydrogel is synthesized by free radical polymerization in an assembled mould. The mould is assembled with a quartz glass plate, a Teflon plate and a Teflon spacer. In the mould, the monomers are polymerized under UV irradiation from the quartz glass side. Due to the attenuation of UV light strength along the irradiation distance, there exists a gradient distribution of crosslinkage of hydrogel networks along the spacer height. Thus, poly(AMPS-*co*-AAm) hydrogels with anisotropic networks can be produced, with the body slightly bending to the more densely crosslinked side in water to form hydrogel walkers with an arc-shape containing two legs. The poly(AMPS-*co*-AAm) hydrogel walkers possess crosslinked networks with negatively-charged sulfonic groups. When applying an electric field to the hydrogel walkers in NaCl solution, the free ions of NaCl solution can move toward the counter-electrodes. However, for the hydrogels, only the mobile counterions of the negatively-charged sulfonic groups can move toward the counter-electrodes, since the motion of the sulfonic groups is restricted by the crosslinked networks. Such ion motions can create an ionic concentration gradient within the hydrogel networks along the direction of the electric field, resulting in an osmotic pressure difference within the hydrogel walkers^14^,^23^. The back side of the hydrogel walker that faces the anode possesses larger osmotic pressure than the belly side that faces the cathode, thus showing more swollen state and leading to bending of the hydrogel walker toward the belly side. As the bending behavior is induced by the ionic gradient that driven by electric field, increase of the applied voltage can lead to faster ion motion for generating larger ionic gradient^34^. Thus, the hydrogel can display faster deformation rate, and larger degree of bending deformation. Moreover, the bending behaviors are reversible and repeatable by repeatedly applying "on/off" electric field. Therefore, the bending/stretching behaviors of the poly(AMPS-*co*-AAm) hydrogels can be well-controlled by regulating the applied voltage. Such bending/stretching behaviors of the poly(AMPS-*co*-AAm) hydrogel walkers enable moving of the two legs on a rough surface to achieve one-directional walking motion under repeated "on/off" electric field. Moreover, although loading cargo suppresses the bending/stretching behaviors, hydrogel walkers that loaded with very heavy cargo still exhibit efficient walking motion for achieving cargo transportation.

In summary, we have developed a simple approach for creation of arched poly(AMPS-*co*-AAm) hydrogel walkers that enable electro-driven motion for cargo transport. The hydrogel walkers allow controllable and reversible bending/stretching behaviors via repeated "on/off" electrical fields in electrolyte solution. Based on such bending/stretching behaviors, the hydrogel walkers can move their two legs to achieve one-directional walking motion on a rough surface via repeated "on/off" electro-triggering cycles. Furthermore, the hydrogel walkers can also achieve one-directional walking motion when loaded with very heavy cargo for efficient cargo transport. The poly(AMPS-*co*-AAm) hydrogel walkers with simple design/fabrication strategy and excellent cargo-loaded walking behaviors are promising for developing electro-controlled soft systems in the soft robotic field for remote manipulation and transportation.

## Methods

### Materials

2-Acrylamido-2-methylpropanesulfonic acid (AMPS) was purchased from Sigma-Aldrich. Acrylamide (AAm), *N*,*N*’-methylenebisacrylamide (MBA) and sodium chloride (NaCl) were purchased from Chengdu Kelong Chemical Reagents. 2-oxoglutaricacid (KGA) was purchased from J&K Scientific Ltd. All chemicals were used as received without further purification. Deionized water (18.2 MΩ, 25 °C) from a Milli-Q Plus water purification system (Millipore) was used throughout this work.

### Fabrication of Poly(AMPS-*co*-AAm) Hydrogel Walkers

The hydrogel walker was synthesized by free radical polymerization. 0.7 M AMPS, 0.3 M AAm, 0.05 M cross-linking agent MBA, and 0.008 M initiator KGA were dissolved in deionized water as monomer solution. Nitrogen gas was bubbled into the monomer solution for 15 min to remove dissolved oxygen in the system. For fabricating hydrogel walkers with looper-like shape, a quartz glass plate, a Teflon plate spacer (thickness: 0.3 mm) and a teflon plate were sequentially assembled by clamps to create a mould for hydrogel synthesis (Fig. 1). Next, the monomer solution was injected into the mould and then converted into hydrogel by UV-irradiation for 20 min. After polymerization, the synthesized hydrogel was taken out of the dissembled mould and then immersed in deionized water for 1 week to remove the residual chemicals. The obtained poly(AMPS-*co*-AAm) hydrogel with thickness of ca. 0.5 mm was cut into uniform strips as the hydrogel walkers for further study. All hydrogel walkers were dyed with methylene blue for easy observation. The chemical composition of the hydrogel walker was characterized by FT-IR (IR Prestige-21, Shimadzu). The crosslinkage of the hydrogel walker was investigated by studying the distribution of sulfur element that contained only in the AMPS monomer at the cross-section of hydrogel walker using SEM (JSM-7500F, JEOL) coupled with Energy Dispersive Spectrometer (EDS).

### Study on the Electro-Induced Bending Behaviors of Poly(AMPS-*co*-AAm) Hydrogel Walkers

The bending behaviors of poly(AMPS-*co*-AAm) hydrogel walkers in response to electric field were investigated by using hydrogel walkers with fully stretched size of 10.0 mm × 2.0 mm × 0.5 mm (length × width × thickness) as the sample. Two parallel carbon electrodes, horizontally placed 44 mm apart, were immersed in 0.01 M NaCl solution to apply the electric field. Then, the hydrogel walker was immersed in the NaCl solution and placed in the centre of the two carbon electrodes, with the belly side facing the cathode (Fig. S4). We have studied the effect of applied voltage on the bending behavior of the hydrogel walkers and the reversible bending/stretching behaviors. The degree of bending deformation (*ε*) was employed to evaluate the bending behaviors, which can be calculated by the following equation:


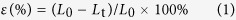


where *L*_o_ is the distance between the two legs of hydrogel walker (Fig. 1f) before applying electric field; *L*_t_ is the distance between the two legs of hydrogel walker at time *t*. The reversible bending/stretching behaviors of the hydrogel walkers were investigated by alternately applying an electric voltage of 25 V to the hydrogel walkers for 10 s and then turning off the electric voltage for 15 s. The bending/stretching behaviors were recorded with a digital camera (E-PL5, Olympus).

### Study on the Electro-Driven Walking Behaviors of Poly(AMPS-*co*-AAm) Hydrogel Walkers For Cargo Transport

The electro-induced walking behaviors of poly(AMPS-*co*-AAm) hydrogel walkers were studied by using hydrogel walkers with fully stretched size of 10.0 mm × 4.0 mm × 0.5 mm as the sample. The distance between the ends of their two legs (*L*_0_) was 8.6 mm. Meanwhile, the fully stretched size of the hydrogel in dry state was 2.6 mm × 1.0 mm × 0.1 mm. The hydrogel walker, with mass (*m*_0_) of 1.058 mg at dried state, was loaded with different weights of Teflon cargo by an adhesive (Konishi). The mass percentages of the loads compared to the dry mass of the hydrogel were 25 *m*_0_, 50 *m*_0_, 75 *m*_0_, 100 *m*_0_, and 125 *m*_0_, respectively. Then, the cargo-loaded hydrogel walker was placed onto a resin ratchet substrate fabricated by Stereo Lithography Apparatus for the walking. Considering that the loaded hydrogel walkers suffer from larger resistance against their movements, two parallel carbon electrodes, both immersed in 0.01 M NaCl solution, were vertically placed 34 mm apart to provide stronger electric intensity for powering their walking. The ratchet substrate was centrally placed between the two parallel carbon electrodes (Fig. 4). The cathode electrode at the bottom was covered with dialysis membrane to prevent the H_2_ gas flow from NaCl solution electrolysis disturbing the walking of hydrogel walker. The walking behaviors of cargo-loaded hydrogel walkers on different sizes of ratchet plates were studied to investigate the effect of ratchet topography on the walking motion. Then, the walking behaviors of hydrogels walkers loaded with different weights of cargo were studied to investigate the effect of cargo weight on the walking motion. All the experiments were conducted at a voltage of 25 V. The walking motions were also recorded with the digital camera.

## Additional Information

**How to cite this article**: Yang, C. *et al.* Hydrogel Walkers with Electro-Driven Motility for Cargo Transport. *Sci. Rep.* **5**, 13622; doi: 10.1038/srep13622 (2015).

## Supplementary Material

Supplementary Information

Supplementary Movie S1

Supplementary Movie S2

Supplementary Movie S3

Supplementary Movie S4

Supplementary Movie S5

Supplementary Movie S6

Supplementary Movie S7

Supplementary Movie S8
