# Supplementary material for: Hydrogel Walkers with Electro-Driven Motility for Cargo Transport
Source: Sci Rep. 2015 Aug 28;5:13622. doi: 10.1038/srep13622 (PMC4551975; doi:10.1038/srep13622)
Supplement: Supplementary Information [file srep13622-s1.doc]

Supplementary Information

**Hydrogel Walkers with Electro-Driven Motility for Cargo Transport**

Chao Yang^1^, Wei Wang^1^, Chen Yao^1^, Rui Xie^1^, Xiao-Jie Ju^1,2^, Zhuang Liu^1^ & Liang-Yin Chu^1,2^

^1^ School of Chemical Engineering, Sichuan University, Chengdu, Sichuan 610065, China

^2^ State Key Laboratory of Polymer Materials Engineering, Sichuan University, Chengdu, Sichuan 610065, China.

**Supplementary Figures S1-S6**

**
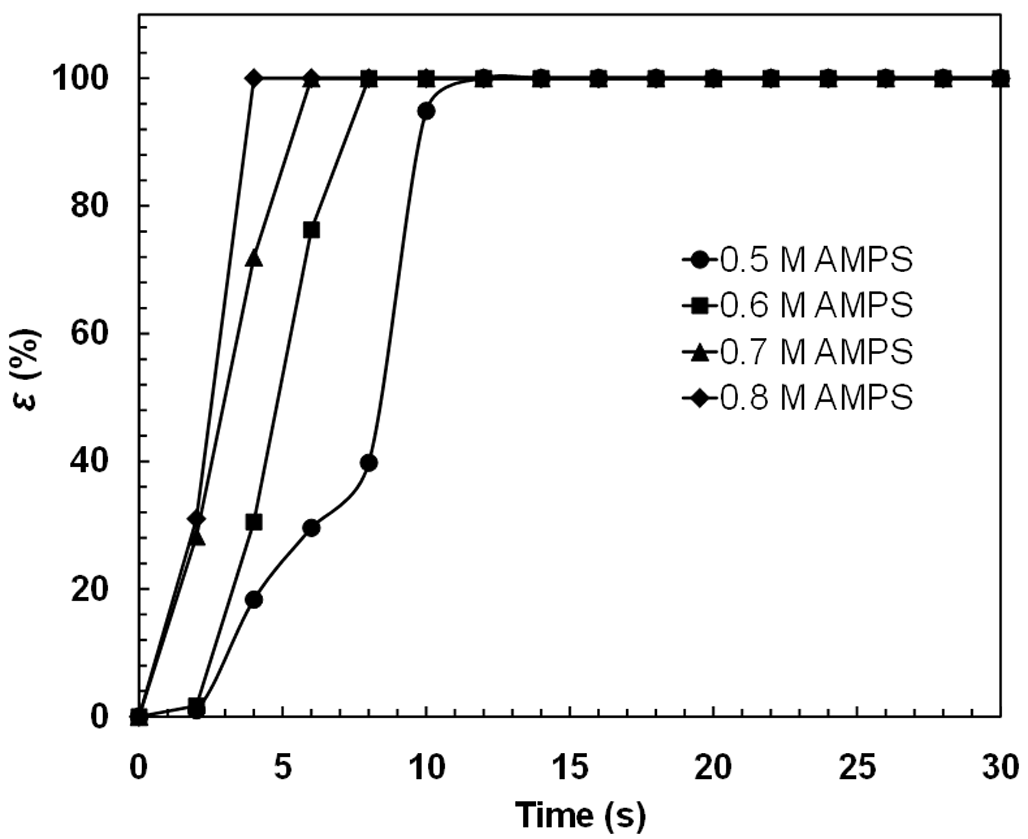
**

**Figure S1 | Effect of the monomer ratio of AMPS and AAm on the electro-responsive property of hydrogel walkers.** The total molarity of AMPS and AAm is fixed at 1 M.

**
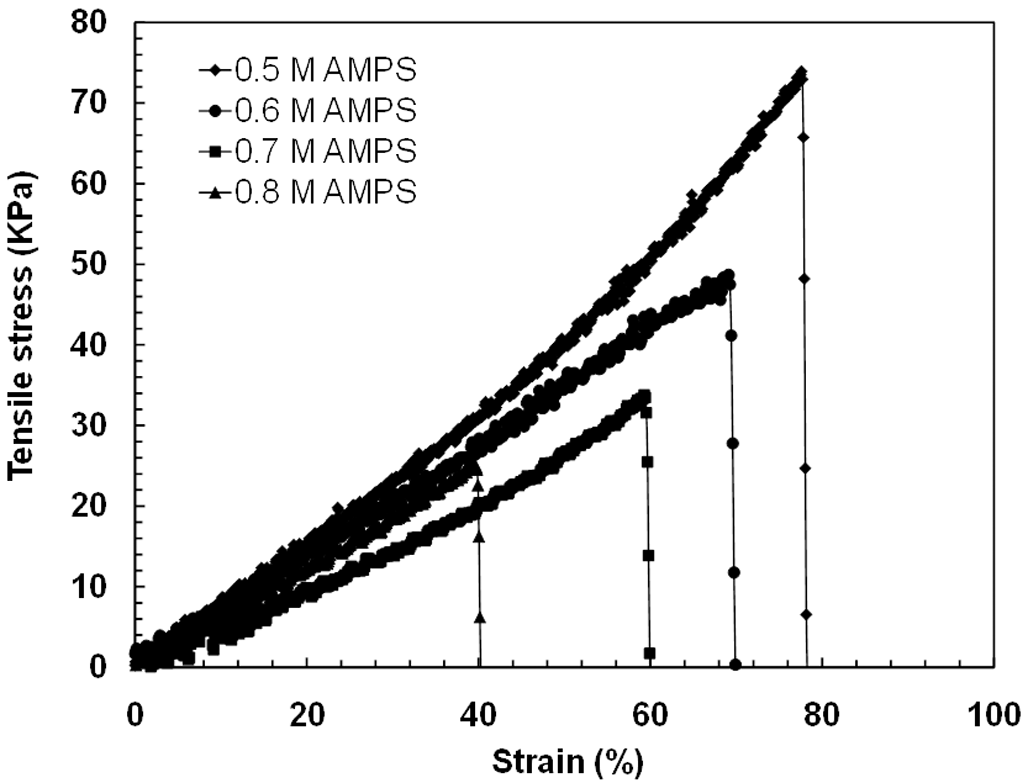
**

**Figure S2 | Effect of the monomer ratio of AMPS and AAm on the mechanical strength of hydrogel walkers.** The total molarity of AMPS and AAm is fixed at 1 M. The mechanical strenth of the hydrogel walker was tested by Tensile machine (EZ-LX, Shimadzu).


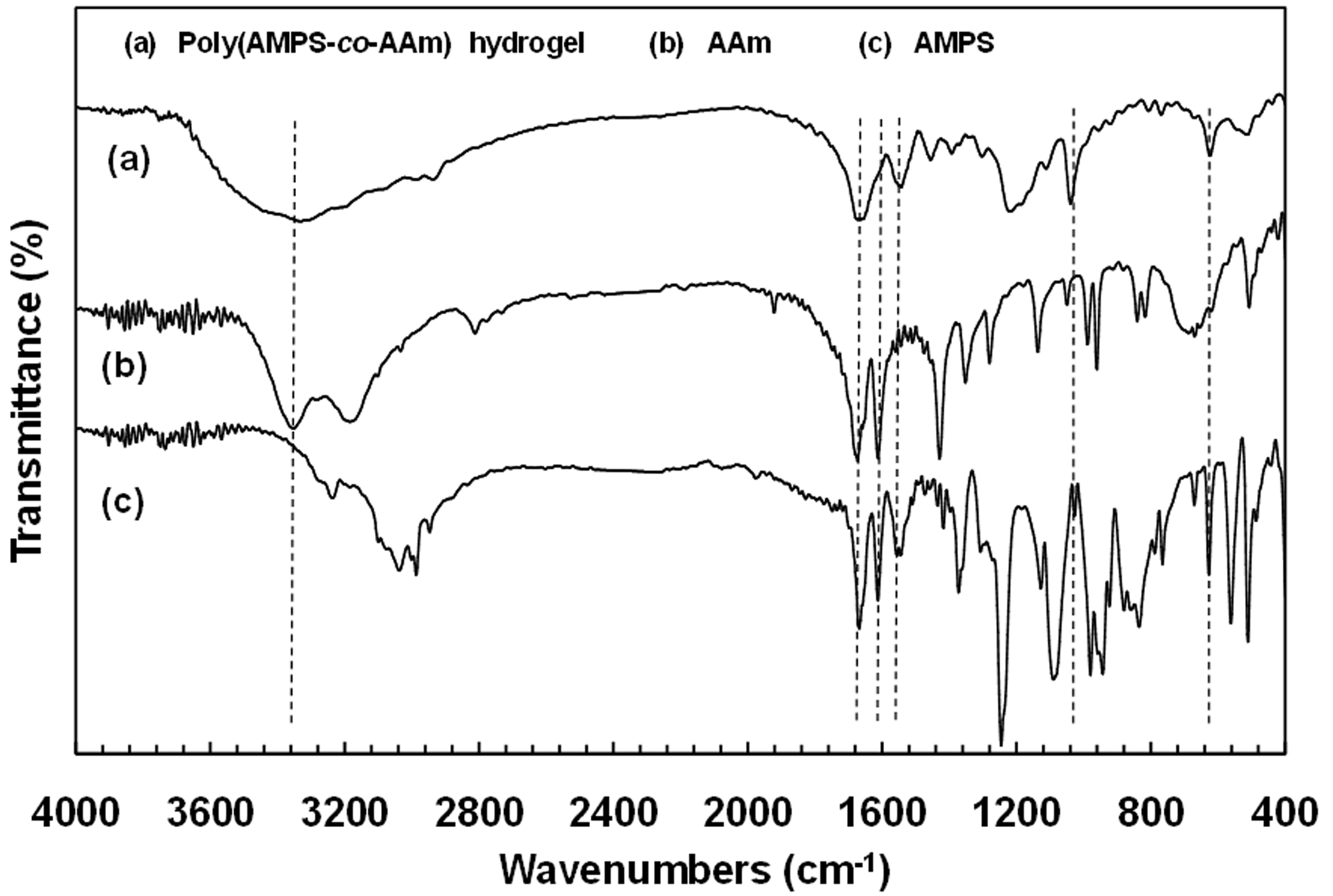


**Figure S3 | FT-IR spectrum of** **poly(AMPS-*co*-AAm) hydrogel (a), AAm (b) and AMPS (c)** In the FT-IR spectra of the poly(AMPS-*co*-AAm) hydrogel, the broad absorption peak at 3350 cm^-1^ is N-H stretching vibration band, the strong peak at 1670 cm^-1^ is C=O stretching vibration band, the peak at 1547 cm^-1^ is N-H bending vibration band, and the peaks at 1040 cm^-1^ and 620 cm^-1^ are the characteristic bands of sulfonic group. The characteristic band of C=C stretching vibration at 1617 cm^-1^ disappears after the free radical polymerization, which indicates the copolymerization and crosslinking of the AMPS and AAm, and confirms the successful fabrication of poly(AMPS-*co*-AAm) hydrogel.


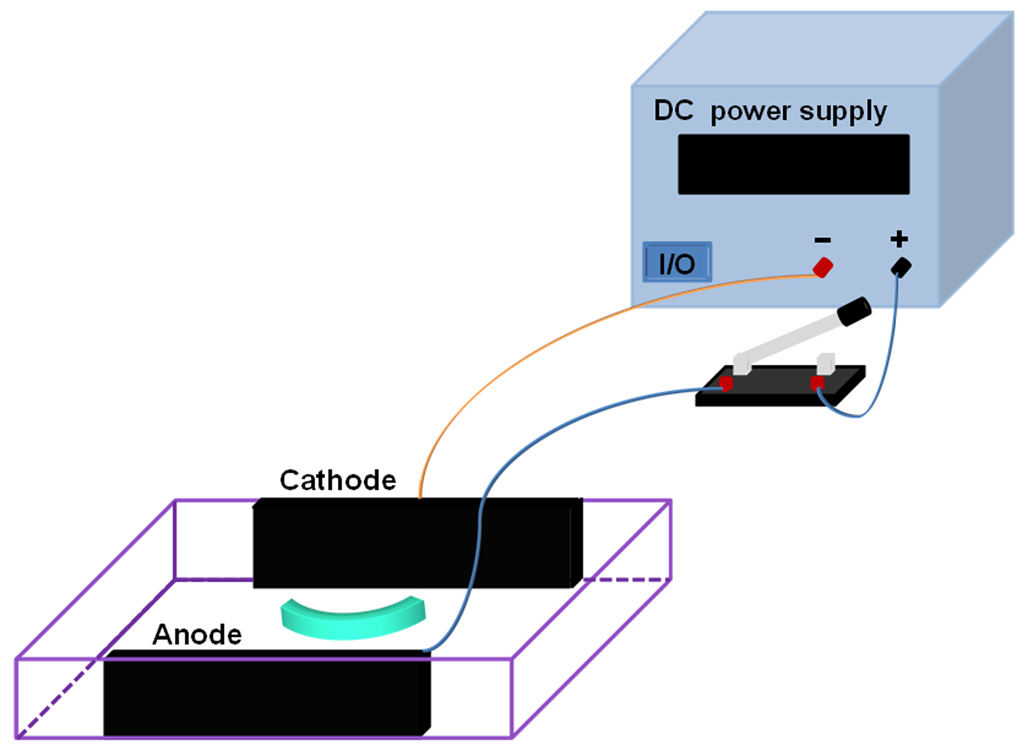


**Figure S4 | Setup for studying the electro-induced bending behaviours of the poly(AMPS-*co*-AAm) hydrogel walkers**


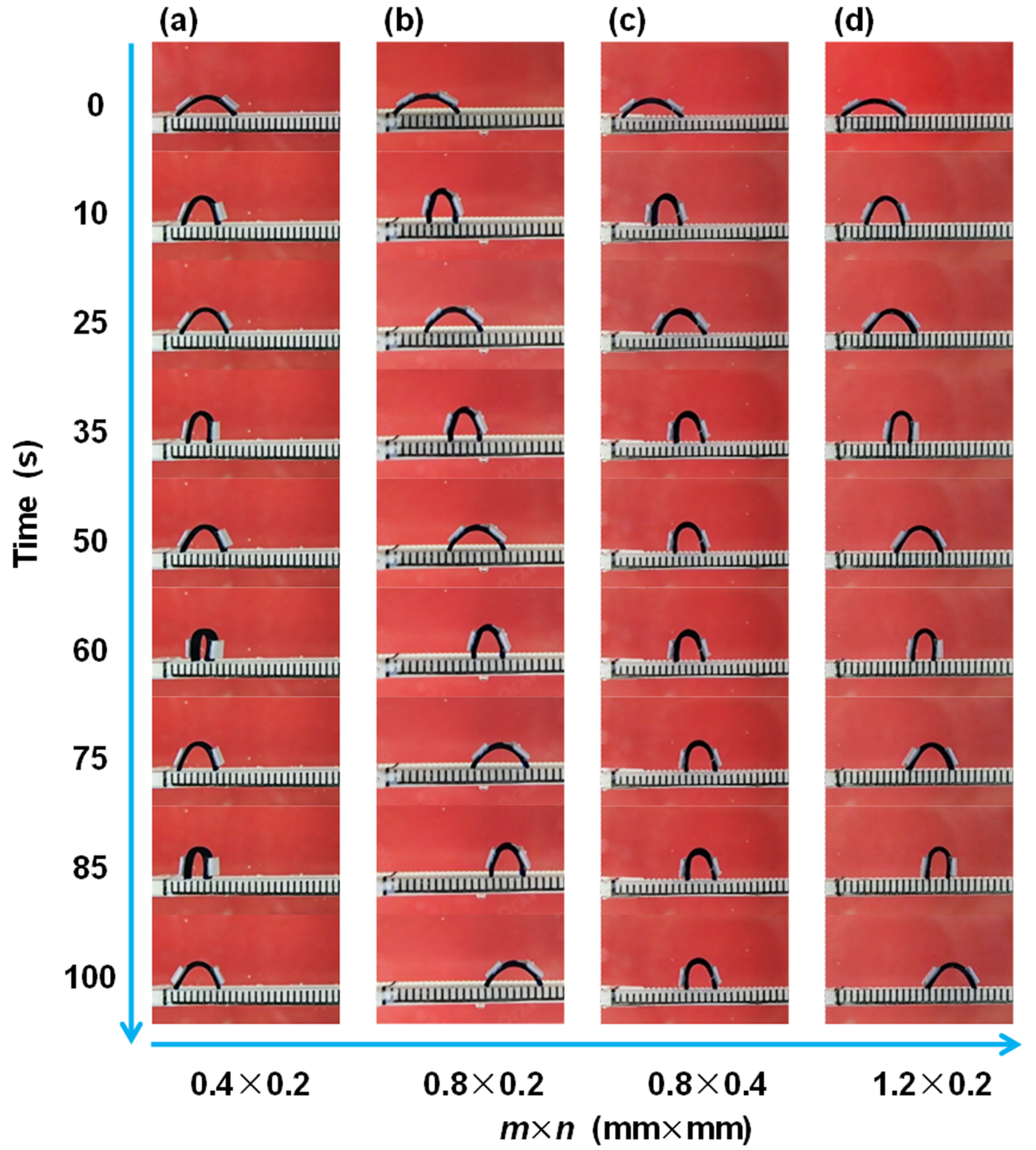


**Figure S5 | Photographs showing** **the walking behaviors of hydrogel walker loaded with cargo of 25 *m*_0_ on ratchet plates with different step sizes**, in which *m*×*n* = 0.4 mm×0.2 mm (a), 0.8 mm×0.2 mm (b), 0.8 mm×0.4 mm (c), and 1.2 mm×0.2 mm (d).


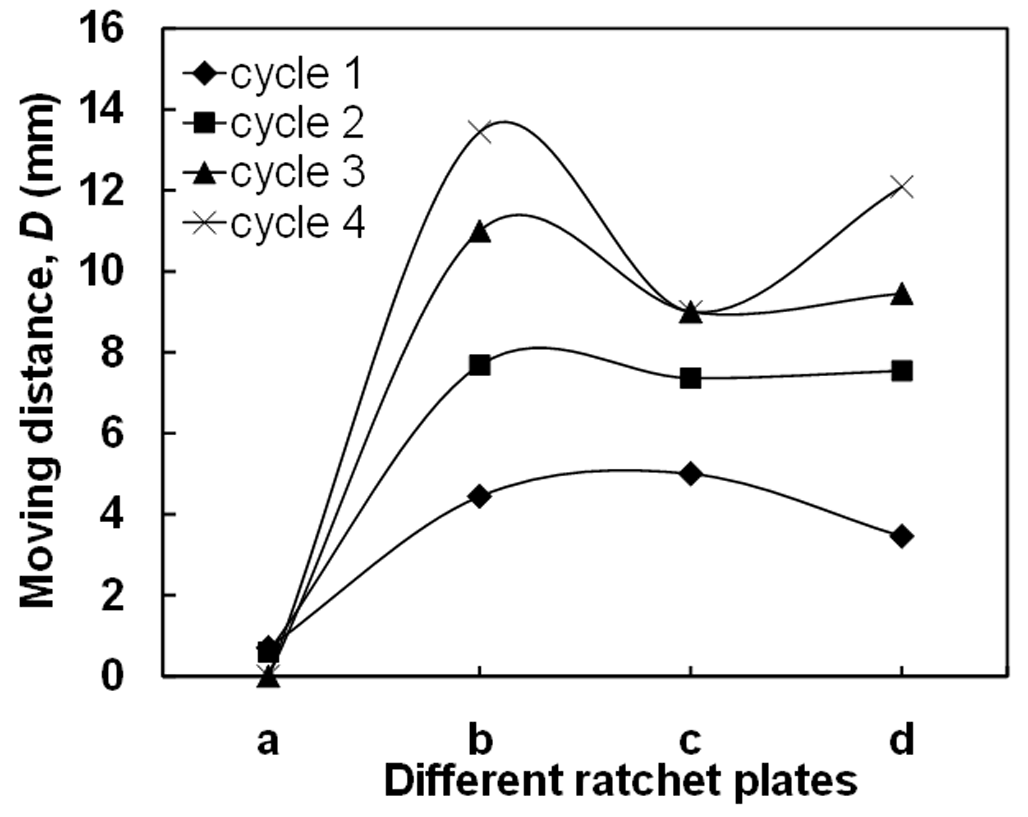


**Figure S6 | Moving distances of the hydrogel walker loaded with cargo of 25 *m*_0_ on ratchet plates with different step sizes**, in which *m*×*n* = 0.4 mm×0.2 mm (a), 0.8 mm×0.2 mm (b), 0.8 mm×0.4 mm (c), and 1.2 mm×0.2 mm (d).

**Supplementary Movies S1-S8**

**Movie S1 | Walking behavior of hydrogel walker loaded with cargo of 25 *m_0_* on ratchet plate with step size of *m*×*n* = 0.4 mm×0.2 mm**

**Movie S2 | Walking behavior of hydrogel walker loaded with cargo of 25 *m_0_* on ratchet plate with step size of *m*×*n* = 0.8 mm×0.2 mm**

**Movie S3 | Walking behavior of hydrogel walker loaded with cargo of 25 *m_0_* on ratchet plate with step size of *m*×*n* = 0.8 mm×0.4 mm**

**Movie S4 | Walking behavior of hydrogel walker loaded with cargo of 25 *m_0_* on ratchet plate with step size of *m*×*n* = 1.2 mm×0.2 mm**

**Movie S5 | Walking behavior of hydrogel walker loaded with cargo of 50 *m_0_* on ratchet plate with step size of *m*×*n* = 0.8 mm×0.2 mm**

**Movie S6 | Walking behavior of hydrogel walker loaded with cargo of 75 *m_0_* on ratchet plate with step size of *m*×*n* = 0.8 mm×0.2 mm**

**Movie S7 | Walking behavior of hydrogel walker loaded with cargo of 100 *m_0_* on ratchet plate with step size of *m*×*n* = 0.8 mm×0.2 mm**

**Movie S8 | Walking behavior of hydrogel walker loaded with cargo of 125 *m_0_* on ratchet plate with step size of *m*×*n* = 0.8 mm×0.2 mm**
